# MED12 Regulates Smooth Muscle Cell Functions and Participates in the Development of Aortic Dissection

**DOI:** 10.3390/genes13040692

**Published:** 2022-04-14

**Authors:** Yingchao Zhou, Lingfeng Zha, Jianfei Wu, Mengru Wang, Mengchen Zhou, Gang Wu, Xiang Cheng, Zhengrong Huang, Qiang Xie, Xin Tu

**Affiliations:** 1 Heart Center, Qingdao Women and Children’s Hospital, Qingdao University, Qingdao 266034, China; zhouyc@qdu.edu.cn; 2 Key Laboratory of Molecular Biophysics of the Ministry of Education, Center for Human Genome Research, College of Life Science and Technology, Huazhong University of Science and Technology, Wuhan 430074, China; wujianfei0309@163.com (J.W.); d201677483@hust.edu.cn (M.W.); 3 Department of Cardiology, Union Hospital, Tongji Medical College, Huazhong University of Science and Technology, Wuhan 430022, China; zhalf@hust.edu.cn (L.Z.); zhmc9990@163.com (M.Z.); nathancx@hust.edu.cn (X.C.); 4 Department of Cardiology, Renmin Hospital of Wuhan University, Wuhan 430060, China; wugangmd@163.com; 5 Department of Cardiology, The First Affiliated Hospital of Xiamen University, Xiamen 361003, China; huangzhengrong@xmu.edu.cn

**Keywords:** aortic dissection, MED12, smooth muscle cell, TGFβ signaling pathway

## Abstract

Aortic dissection (AD) is a life-threatening disease with high morbidity and mortality, and effective pharmacotherapeutic remedies for it are lacking. Therefore, AD’s molecular pathogenesis and etiology must be elucidated. The aim of this study was to investigate the possible mechanism of mediator complex subunit 12 (human: *MED12*, mouse: *Med12*)involvement in AD. Firstly, we examined the expression of MED12 protein (human: MED12, mouse: Med12) in the aortic tissues of AD patients and AD mice. Subsequently, *Med12* gene silencing was accomplished with RNA interference (siRNA). The effects of Med12 on AD and the possible biological mechanisms were investigated based on the proliferation, senescence, phenotypic transformation, and its involved signal pathway of mouse aortic smooth muscle cells (MOVAS), s. The results show that the expression of MED12 in the aortae of AD patients and AD mice was decreased. Moreover, the downregulation of Med12 inhibited the proliferation of MOVAS and promoted senescence. Further research found that Med12, as an inhibitor of the TGFβ1 signaling pathway, reduced the expression of Med12 and enhanced the activity of the TGFβ1 nonclassical signaling pathway, while TGFβ1 inhibited the phenotype transformation and proliferation of MOVAS by inhibiting Med12 synthesis. In conclusion, Med12 affected the phenotype, proliferation, and senescence of MOVAS through the TGFβ signaling pathway. This study provides a potential new target for the prevention and treatment of AD.

## 1. Introduction

AD is defined as a life-threatening arterial disease characterized by the tearing of the intima of the aorta, in which blood enters the medial layer, resulting in the formation of true and false lumens in the aorta [1]. Since the thoracic aorta is vulnerable to rupture, AD has high morbidity and mortality rates [2]. The current clinical management of AD is mainly achieved through surgical intervention, and there are few effective pharmacotherapeutic regimens available [3,4,5], which makes the search for novel therapeutic targets valuable.

AD is mediated by vascular smooth muscle cell (VSMC) remodeling [6], increased vascular inflammation [7], and extracellular matrix degradation [8]. VSMCs play a key role in maintaining vascular function and homeostasis. In particular, it has been proposed that the abnormal phenotypic transformation, migration, proliferation, and contractile dysfunction of the VSMC promote the progression of AD [9,10]. Because phenotypic changes in the VSMC control the aortic structure and function, studies have focused on understanding the regulation of VSMC phenotypes and identifying the factors and pathways involved. Transforming growth factor-β (TGFβ) signaling is a key regulatory pathway in maintaining the aortic contractile phenotype and function [11,12]. TGFβ stimulation results in the decreased expression of VSMC proteins (e.g., SM22α and α-SMA) and the increased expression of inflammatory proteins (e.g., matrix metalloproteinase (MMP)-2 and MMP-9) [13,14,15]. However, it is not yet completely understood whether VSMC homeostasis is associated with vascular function and AD progression.

As a major functional component of the mediator complex, MED12 is highly conserved. It interacts with transcription factors for gene-specific transcription [14]. Molecular genetic studies have demonstrated that the MED12 germline mutation can cause familial inherited diseases, and some patients have shown vascular structural abnormalities (e.g., aneurysm) [16,17,18], suggesting that MED12 is one of the most important genes involved in cell proliferation [19], differentiation [20], and development of organs [21].

In the present study, the expression of MED12 was examined in the aortic tissues of AD and normal (non-AD) patients. We then found a similar expression pattern of Med12 in AD and control mice. Subsequently, the effects of Med12 on AD and possible biological mechanisms were investigated through cell biology experiments, such as proliferation, senescence, and phenotypic transformation of mouse aortic smooth muscle cells (MOVAS), and these all involved signal pathways. This study indicates that the MED12 might be involved in AD formation and serve as a promising therapeutic target for AD.

## 2. Materials and Methods

### 2.1. Human Samples

Human aortic tissue samples were collected from patients undergoing surgical repair of aortic dissection (*n* = 3). Abandoned aortae after heart transplantation were used as control aortic tissues (*n* = 3). One part of the tissue was stored at −80 °C for RT-PCR detection, and another part was stored in 4% paraformaldehyde solution for further analysis. The present study was approved by the Ethics Committee of Huazhong University of Science and Technology and adhered to the guidelines of the Declaration of Helsinki. Written informed consent was obtained from each participant.

### 2.2. Construction of the AD Mice Model

C57BL/6 (4-week-old, male) mice were randomly divided into control (*n* = 10) and experimental (*n* = 10) groups. Mice in the control and experimental groups were fed a normal diet for 4 weeks, and the drinking water of the experimental group contained fresh β-aminopropionitrile (BAPN, Sigma-Aldrich, St Louis, MO, USA, 0.6%) [22,23]. During the experiment, the mice were housed at 22 ± 1 °C, 55% RH, and a 12 h:12 h dark–light cycle. After the mice were sacrificed, the aorta was separated and stored in 4% paraformaldehyde for further analysis. Animal studies were approved by the Huazhong University of Science and Technology Animal Care and Use Committee and performed in accordance with the Guide for the Care and Use of Laboratory Animals (https://www.nap.edu/catalog/12910/guide-for-the-care-and-use-of-laboratory-animals-eighth)[Accessed: 21 January 2020].

### 2.3. Immunohistochemistry

The tissues were embedded in paraffin. MED12 expression was determined by standard immunohistochemical protocol. The tissue sections were incubated with MED12 primary antibody (0.5 µg/mL, 20028-1-AP, Proteintech, Wuhan, China), and immunohistochemical controls were incubated with rabbit IgG (8 µg/mL, ab172730Abcam, Cambridge, MA, USA,) instead of the primary antibody. Then, slides were incubated with secondary antibodies (0.2 µg/mL, GB23303, Servicebio, Wuhan, China). The section was scanned and photographed, and 3 fields were selected for each sample. The mean integrated optical density (IOD) of positively stained tissue in the selected field was measured using Image J software (version 1.53c).

### 2.4. Immunofluorescence

Tissue sections were prepared as described above. For double immunofluorescence, the MED12 (2 µg/mL, 20028-1-AP, Proteintech, Wuhan, China) and α-SMA (1.5µg/mL, 14395-1-AP, Proteintech, Wuhan, China ) primaries were co-incubated and subsequently labeled with fluorescenated secondary antibodies (0.01–0.02 µg/mL, Servicebio, Wuhan, China). Nuclei were stained with DAPI (0.5 µg/mL, Beyotime, C1002, Shanghai, China). Sections were examined via immunofluorescence microscopy (Nikon E100, Tokyo, Japan) and photographed. Three visual fields of each section were randomly selected, and the average fluorescence intensity was calculated by ImageJ software (version 1.53c). Colocalization analysis of immunofluorescence images was performed with ImageJ software using the colocalization plugin Coloc2, and Pearson’s correlation coefficient (PCC) was employed to quantify colocalization.

Cell immunofluorescence was performed as described previously [24]. The SM22α (1.5 µg/mL, 10493-1-AP, Proteintech, Wuhan, China) and α-SMA (1.5 µg/mL, 14395-1-AP, Proteintech, Wuhan, China) primaries were co-incubated and subsequently labeled with fluorescenated secondary antibodies (0.01–0.02 µg/mL, Servicebio, Wuhan, China). Nuclei were stained with DAPI (0.5 µg/mL, C1002, Beyotime, Shanghai, China). The quantification of the mean fluorescence intensity was performed using ImageJ Software (version 1.53c) for three images/samples. Cellular aspect ratio calculations (size index) were measured by dividing the longest length by the widest width in ImageJ software (version 1.53c).

### 2.5. Cell Culture

Mouse aortic smooth muscle cells (MOVAS) were purchased from the American Type Culture Collection (Manassas, VI, USA). The MOVAS were passaged periodically and sub-cultured to 80% confluence. Early passage cells (fewer than 20 passages) were used for the study and discarded after 20 passages. Cells were seeded in Dulbecco’s modified Eagle’s medium (DMEM) supplemented with 10% fetal bovine serum (FBS) and cultured in a humidified incubator at 37 °C under a 5% CO_2_ atmosphere.

### 2.6. RNA Interference (siRNA)

MOVAS suspensions were placed in a 6-well plate that had been pre-incubated for 24 h. MOVAS with densities of 50–60% were transfected with Med12 siRNA (5′-GCCAUGCCAUCAAGAAGAUTTAUCUUCUUGAUGGCAUGGCTT-3′) or control siRNA (5′-UUCUCCGAACGUGUCACGUTTACGUGACACGUUCGGAGAATT-3′) for 72 h via Lipofectamine 2000 (Invitrogen, Carlsbad, CA, USA) according to the manufacturer’s instructions. Cells were maintained in 10% FBS-containing medium for the duration of the experiment.

### 2.7. Western Blot Analyses

MOVAS were treated as described in Section 2.6, and then transfected with siRNA or incubated with TGFβ1 (4 ng/mL) for 72 h. MOVAS were maintained in the 10% FBS-containing medium for the duration of the experiment. Cells were then harvested for Western blot. 

Firstly, MOVAS were washed twice with PBS prior to sample preparation. RIPA lysis solution (P0013B, Beyotime, Shanghai, China) was used to lyse the MOVAS at 4 °C for 30 min. Then, SDS–PAGE loading buffer (P0015F, Beyotime, Shanghai, China) was added to the lysate, and the mixture was boiled at 98 °C for 15 min. A BCA kit (PC0020, Solarbio, Beijing, China) was used to detect total protein concentration, and 20–30 µg of protein per sample was separated with SDS–PAGE gels. Next, proteins were transferred onto polyvinylidene fluoride (PVDF) membranes (Millipore, Billerica, MA, USA, ISEQ00010). The blots were incubated overnight at 4 °C with the corresponding primary antibodies and incubated with secondary antibodies at room temperature for 2 h. ChemiDoc XRS (Bio-Rad Laboratories, Hercules, CA, USA) was used to detect the signals. Quantity One Basic v. 4.4.0 (Bio-Rad Laboratories, Hercules, CA, USA) was used to quantify the band intensities. 

The antibodies used in this study included GAPDH (0.3 µg/mL, G9454, Sigma-Aldrich, St Louis, MO, USA), α-Tubulin (0.5 µg/mL, T6199, Sigma-Aldrich, St Louis, MO, USA), SM22α (0.3 µg/mL, 10493-1-AP, Proteintech, Wuhan, China), MED12 (0.9 µg/mL, 20028-1-AP, Proteintech, Wuhan, China), α-SMA (0.5 µg/mL, 14395-1-AP, Proteintech, Wuhan, China), PCNA (0.1 µg/mL, 13110, CST, Danvers, MA, USA), SMAD2 (0.3 µg/mL, 5339, CST, Danvers, MA, USA), SMAD3 (0.2 µg/mL, 9523, CST, Danvers, MA, USA), Phospho-SMAD2 (0.05 µg/mL, 3108, CST, Danvers, MA, USA), Phospho-SMAD3 (1.0 µg/mL, 9520, CST, Danvers, MA, USA), Phospho-ERK (0.2 µg/mL, 9101S, CST, Danvers, MA, USA), TGFβR2 (2 µg/mL, abs135929, absin, Shanghai, China), and mouse and rabbit secondary antibodies (0.01–0.02 µg/mL, Servicebio, Wuhan, China,).

### 2.8. CCK-8 Cell Viability Assay

MOVAS suspensions were placed in a 96-well plate that had been pre-incubated for 24 h. MOVAS were synchronized for 12 h in serum-free medium, and the density was 40–50%. MOVAS were transfected with Med12 siRNA or control siRNA for 72 h with six repetitions per concentration. Transfected cells were maintained in medium containing 10% FBS. Analysis of cell viability was performed using a CCK-8 kit (Dojindo, Kumamoto, Japan) following the manufacturer’s protocol. The plates were incubated for 2 h at 37 °C, and absorbances were measured at 450 nm using a microplate reader (MultiSkan, Thermo Fisher Scientific, Waltham, MA, USA).

### 2.9. RT-PCR Assay

MOVAS were treated as described in Section 2.6. Three parallel holes were set for each group. Cells were lysed with TRIzol reagent (TaKaRa, Dalian, China) to extract total RNA. The frozen human aorta tissues were homogenized and then lysed with TRIzol reagent. Then, the total RNA was reverse-transcribed into complementary DNA (cDNA) with the HiScript III 1st Strand cDNA Synthesis Kit (R312-01, Vazyme, Nanjing, China). Quantitative reverse-transcription polymerase chain reaction (RT-PCR) analysis was carried out using Taq Pro Universal SYBR qPCR Master Mix (R712-02, Vazyme, Nanjing, China). PCR primers were designed using GeneTools, and the quality of primers was checked using NCBI-Primer-BLAST. The RT-PCR primer sequences are shown in Table 1. 

### 2.10. β-Galactosidase Staining

MOVAS were seeded in 6-well dishes and transfected with si-Med12 or si-control for 72 h. MOVAS were washed twice with PBS and fixed in 4% paraformaldehyde for 30 min at room temperature. β-galactosidase staining was performed with a senescence-associated β-Galactosidase Staining Kit (Beyotime, Shanghai, China). Finally, MOVAS were photographed with an inverted light microscope (Olympus, Tokyo, Japan), and 7–10 fields of view were chosen for each well. X-gal-positive cells were manually counted.

### 2.11. Statistical Analysis

Data are presented as means ± SD and were analyzed by a two-tailed Student’s *t*-test. *p*-values < 0.05 were considered significant, and the statistical tests and sample size (*n* values) used in the experiments are specified in the figure legends. In the figures, asterisks indicate the *p*-values: * *p* < 0.05, ** *p* < 0.005, and *** *p* < 0.001. GraphPad Prism 6.0b (GraphPad software, La Jolla, CA, USA) was used for the statistical analyses.

## 3. Results

### 3.1. MED12 Was Downregulated in Aortic Dissection (AD)

**Figure 1:**
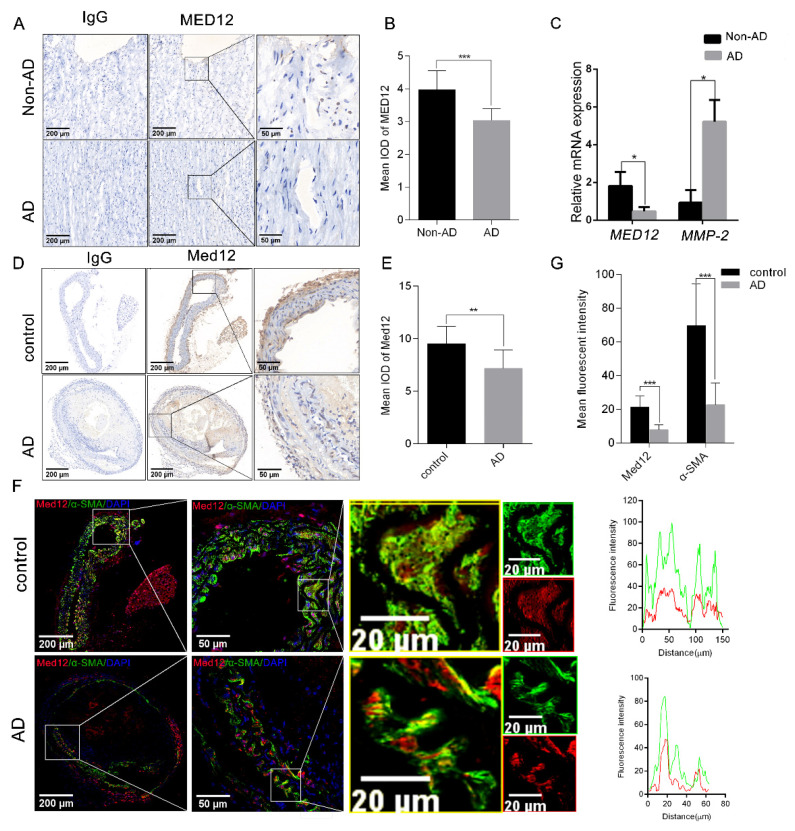
The expression of MED12 in aortic tissue. (**A**) Representative images of immunohistochemical staining in aortic sections from non-AD and AD patients. IgG as a negative control. (**B**) Quantitative analysis of MED12. (**C**) The mRNA expressions of *MED12* in aortae of non-AD and AD patients were detected by RT-PCR, with *MMP-2* as a positive control. (**D**) Representative images of immunohistochemical staining in aortae of AD and control mice. IgG as a negative control. (**E**) Quantitative analysis of Med12. (**F**) Representative images of immunofluorescent staining of Med12 in aortic sections from AD and control mice. Fluorescence intensities of MED12 (red) and α-SMA (green, smooth muscle cell marker) were analyzed. Right insets represent high-magnification images of each individual imaging channel and a merged image of the region (left). Pearson’s correlation coefficient (PCC) was employed to quantify colocalization. (**G**) Quantitative analysis of MED12 and α-SMA fluorescence intensity. Data are expressed as the mean ± SD. Statistical analyses, unpaired *t*-test. * *p* < 0.05; ** *p* < 0.01; *** *p* < 0.001.

To determine the relationship between MED12 and AD, the expression of MED12 was examined in the aortic tissues of AD (*n* = 3) and non-AD (*n* = 3) patients. Immunohistochemistry revealed that the expression of MED12 in AD aorta tissues decreased by 23.5% (*p* < 0.001) compared with non-AD aorta tissues (Figure 1A,B). These results are in accordance with the mRNA *MED12* decreasing by 78% (*p* < 0.05) in AD aorta tissues. Meanwhile, the mRNA levels of *MMP-2* (a positive control) [24], which were increased in aortae from a BAPN-induced AD model, were significantly upregulated (~5-fold, *p* < 0.05) in AD aorta tissues (Figure 1C). We next generated an AD mice model and found a similar expression pattern of Med12 in control and AD mice. Immunohistochemical staining showed that AD aorta had 40.28% (*p* < 0.01) lower Med12 expression than the control (Figure 1D, E). Notably, immunofluorescence staining suggested that Med12 co-located with α-SMA (smooth muscle cell marker), in both AD aorta (PCC = 0.87) and control aorta (PCC = 0.84) (Figure 1F). In addition, consistently with the reduced expression of Med12, α-SMA was reduced by 67.43% (*p* < 0.01) (Figure 1F,G). Collectively, these results suggest that MED12 may be involved in AD by regulating VSMC.

### 3.2. Decreased Expression of Med12 Inhibits Proliferation of Mouse Aortic Smooth Muscle Cells (MOVAS)

**Figure 2:**
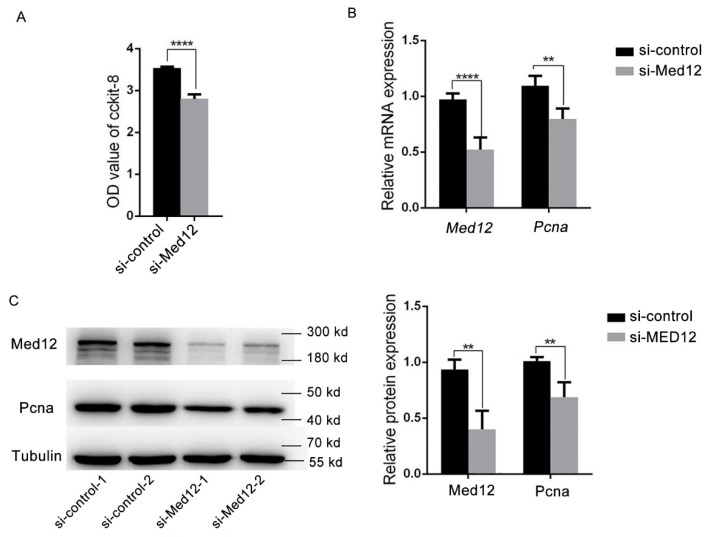
Decreased MED12 inhibited the proliferation of MOVAS. MOVAS transfected with si-control or si-Med12 for 72 h. (**A**) Cell viability detected by CCK-8. (**B**) Expression of *Med12* and *Pcna* detected by RT-PCR. (**C**) Expression of MED12 and Pcna detected by Western blot. The experiment was repeated at least three times. Data are expressed as mean ± SD. Statistical analyses, unpaired *t*-test. ** *p* < 0.01; **** *p* < 0.0001.

Based on the above results, mouse aortic smooth muscle cells (MOVAS) were used as the research subject. MOVAS were transfected with si-control or si-Med12 for 72 h. Compared with the si-control, the cell viability of si-Med12 decreased by 30% (*p*  <  0.0001) due to the CCK8 assay (Figure 2A). The expression of proliferating cell nuclear antigen (Pcna, as a cell proliferation marker) decreased by 25% (*p* < 0.0001) (Figure 2B) and 32% (*p*  <  0.0001) (Figure 2C) in mRNA and protein levels, respectively. These results suggest that the decreased expression of Med12 inhibited the proliferation of MOVAS.

### 3.3. The Decreased Expression of Med12 Promotes the Senescence of MOVAS

**Figure 3:**
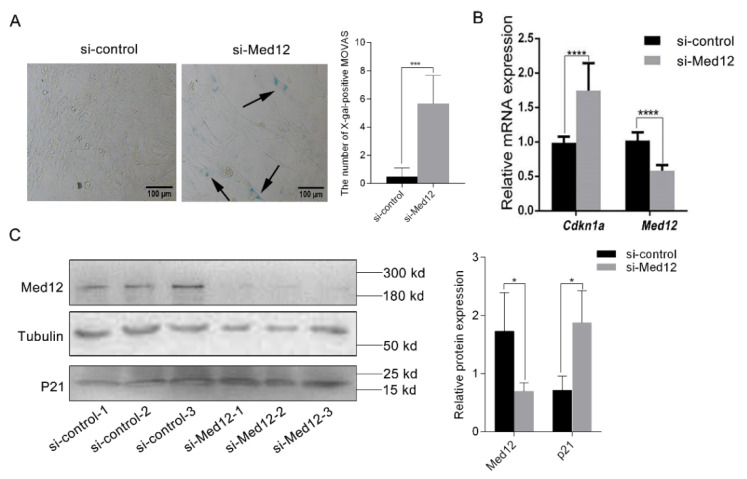
The decreased expression of Med12 promoted the senescence of MOVAS. MOVAS were transfected with si-control or si-Med12 for 72 h. (**A**) Senescent cells were stained with blue by β-galactosidase staining kit as shown by the arrow, and the number of positive cells was quantitatively analyzed. (**B**) The expressions of *Med12* and *Cdkn1a* were detected by RT-PCR. (**C**) The expressions of Med12 and P21 were detected by Western blot. The experiment was repeated at least three times. Data are expressed as mean ± SD. Statistical analyses, unpaired *t*-test. * *p* < 0.05; *** *p* < 0.001; **** *p* < 0.0001.

MOVAS were transfected with the si-Med12 or si-control for 72 h and with β-galactosidase staining assay to examine the status of cell senescence. Compared with the si-control group, the number of X-gal-positive MOVAS increased by 12.29-fold (*p* < 0.001) in the si-Med12 group, suggesting that the decreased expression of Med12 accelerated the senescence of MOVAS (Figure 3A). Next, we measured the expression of *Cdkn1a* (encoding mouse P21 protein), which is involved in the regulation of cell senescence [25]. In the si-Med12 group, the mRNA expression of *Cdkn1a* increased by 1.7-fold (*p* < 0.0001) (Figure 3B), while the expression of P21 increased by 2.63-fold (*p* < 0.05) (Figure 3C). The results show that Med12 affected cell senescence by promoting P21 expression.

### 3.4. Downregulation of Med12 Suppressed Phenotypic Transformation of MOVAS

**Figure 4:**
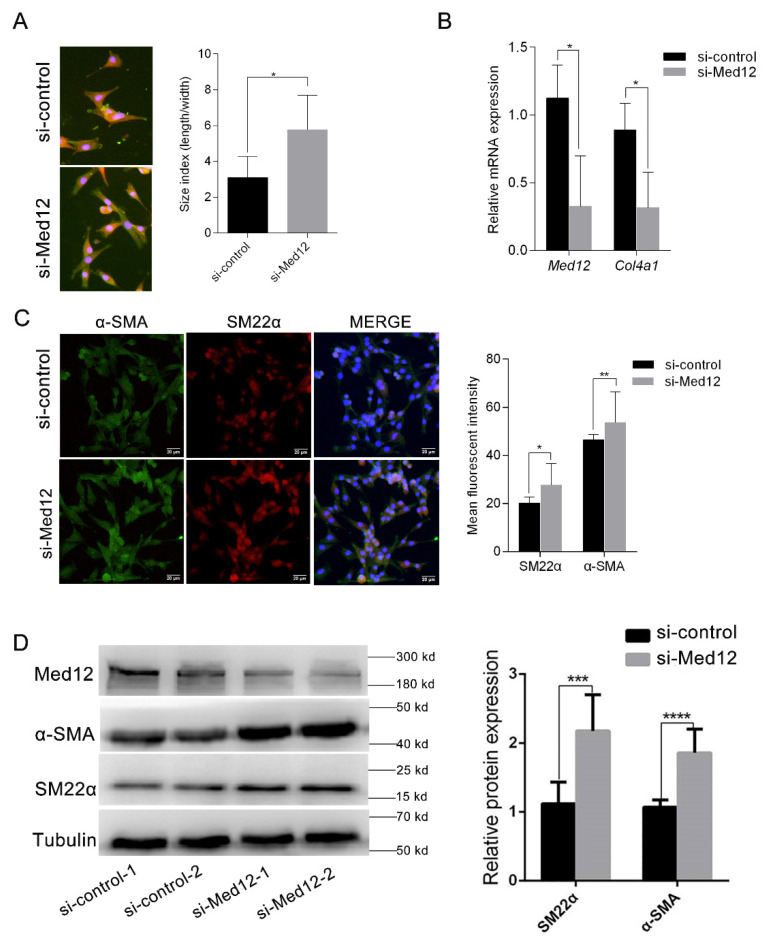
Regulation of cell phenotypic conversion. MOVAS were translated with si-control or si-Med12 for 72 h. (**A**) Fluorescence images were taken using a 40 × objective. The length-to-width ratios of the cells were quantified using ImageJ software. (**B**) Expressions of *col4a1* (encoding collagen IV, synthetic VSMC marker) were detected by RT-PCR. (**C**) Immunofluorescent staining for DAPI (blue), α-SMA (green), and SM22α (red); MERGE represents the combined image of the above three channels. Scale: 20 μm. Statistics of the mean fluorescence in each visual field (4 visual fields/sample, 3 samples/group). (**D**) The expressions of α-SMA and SM22α (contractile VSMC marker protein) were detected by Western blot. The experiment was repeated at least three times. Data are expressed as mean ± SD. Statistical analyses, unpaired *t*-test. * *p* < 0.05; ** *p* < 0.01; *** *p* < 0.001; **** *p* < 0.0001.

After MOVAS were transfected with si-control or si-Med12 for 72 h, the downregulation of Med12 generated clear alterations in cell morphology. The si-Med12 group developed filiform extensions, visualized by an increase in the length-to-width ratio compared to the si-control group (length/width; 5.76 vs. 3.09; *p* < 0.05) (Figure 4A). Meanwhile, compared with the si-control group, the expression of *Clo4a1* (the marker of the synthetic phenotype) decreased by 35.55% (*p* < 0.05) in the si-Med12 group (Figure 4B). These morphometric changes are consistent with a contractile phenotype. α-SMA and SM22α, as marker proteins, were highly expressed in contractile vascular smooth muscle cells [11]. Immunofluorescent staining revealed that α-SMA and SM22α in the si-Med12 group were upregulated by 15.45% (*p* < 0.05) and 27.14% (*p* < 0.01), respectively (Figure 4C). Accordingly, the Western blot showed that SM22α and α-SMA increased by 1.9-fold (*p* < 0.01) and 1.7-fold (*p* < 0.001) (Figure 4D), respectively, in the si-Med12 group compared to the si-control group. These results suggest that the phenotypic transformation of MOVAS was inhibited by the downregulation of Med12 expression.

### 3.5. Effect of MED12 on TGFβ Signaling Pathway

**Figure 5:**
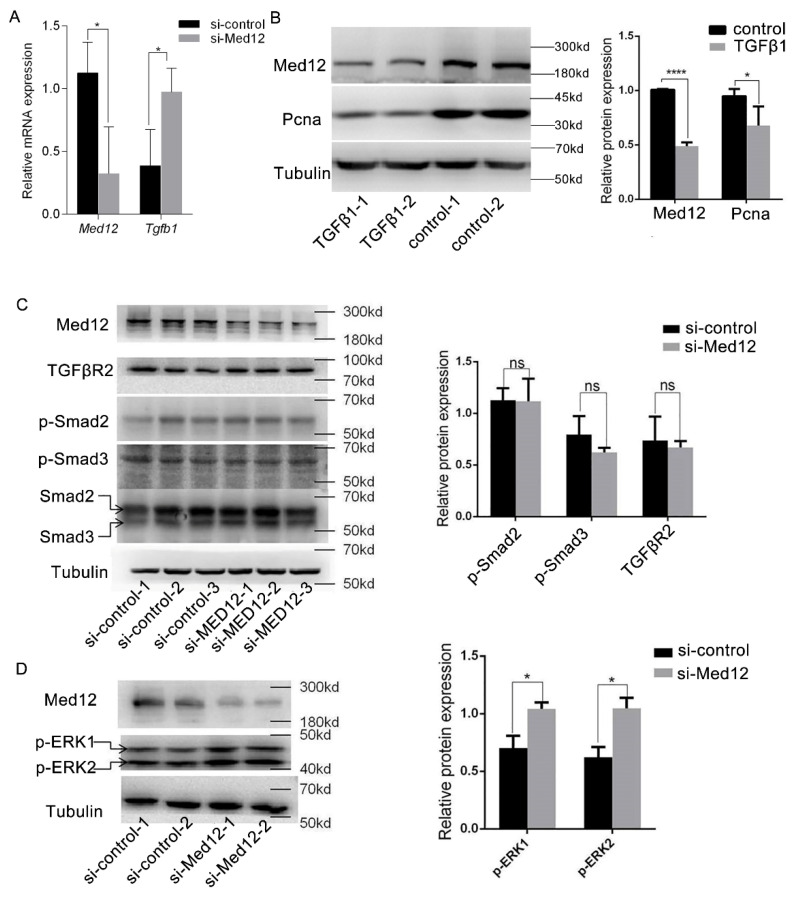
Effect of Med12 on the TGFβ signaling pathway. (**A**) MOVAS were translated with si-control or si-Med12 for 72 h, and the expressions of *Tgfβ1* were detected by RT-PCR. (**B**) MOVAS were incubated with TGFβ1(4 ng/mL) for 72 h, and Western blot detected the expression of Med12 and Pcna. (**C**) Same experiment as in (**A**); the TGFβR2-phosphorylated Smad2 (p-Smad2) relative to total Smad2 and phosphorylated Smad3 (p-Smad3) relative to total Smad3, as detected by Western blot. (**D**) Same experiment as in (**A**), Western blot was used to analyze p-ERK/ERK. The experiment was repeated at least three times. Data are expressed as mean ± SD. Statistical analyses, unpaired *t*-test. * *p* < 0.05; **** *p* < 0.0001. ns: not significant.

MOVAS were translated with si-control or si-Med12 for 72 h. As shown in Figure 5A, the downregulation of Med12 increased TGFβ1 by 2.51-fold (*p* < 0.05), suggesting that Med12 might be involved in the TGFβ signaling pathway. Next, MOVAS were incubated with TGFβ1 (4 ng/mL) [26] for 72 h. Western blot indicated that the expression of Med12 and Pcna decreased by 50% (*p* < 0.0001) and 30% (*p* < 0.05) (Figure 5B), respectively. Furthermore, siRNA was used to knock down Med12 expression, and it detected the TGFβ receptor (TGFβR2) expression and phosphorylation of R-Smads (Smad2, Smad3) in the downstream classical pathway and phosphorylation of ERK1/2 in the nonclassical pathway. Western blot showed that TGFβR2 and phosphorylation of Smad2 and Smad3 did not significantly change after inhibiting the expression of Med12 in MOVAS (Figure 5B). However, the phosphorylated ERK1/2 protein was increased (Figure 5C).

These results suggest that Med12, as an inhibitor of the TGFβ1 signaling pathway, which reduces the expression of Med12, enhanced the activity of the TGFβ1 nonclassical signaling pathway, while TGFβ1 inhibited the phenotype transformation and proliferation of MOVAS by inhibiting Med12 expression.

## 4. Discussion

AD is a life-threatening aortic disease. According to the latest report released by the International Registry of Aortic Dissection (IRAD), the mortality for AD is 35.8%. Of this 35.8%, AD is caused by genetic factors in 25% of patients, and most of the pathogenic genes are autosomal-dominant. Therefore, men and women are theoretically at the same risk of developing AD [27]. However, there are gender differences in AD presentation, age at onset, and outcomes. In a cohort of 965 patients (511 men and 454 women) with aortic aneurysms, Détaint D et al. found that arterial enlargement occurred in 57% of men and 50% of women at age 30, suggesting that aortic enlargement occurs earlier in men than in women [28]. Even when genetic factors are ruled out, men (65%) are more likely to be affected than women [29]. Based on the above results, male mice were used as the research subject in this study. As there are no convincing studies on gender differences in AD animal models, we consider using both male and female mice in future experiments to explore the effect of gender.

The number and phenotypes of VSMC have a great influence on vascular remodeling. A decrease in the number of VSMCs in the aortic middle wall caused by apoptosis is an early marker of the development of AD. With regard to TAV-associated aneurysms, Christian Stern et al. observed a 35% reduction in the number of smooth muscle cells in patients [30]. A decreased number of VSMCs weakens the aortic wall and limits matrix repair capacity. Under physiological conditions, intra-aortic VSMCs mainly exist in contractile form and maintain the normal function of the aorta. Multiple studies have shown that the phenotypic transformation of VSMCs from contractile to syntactic is involved in the occurrence of AD and plays an important role in the pathophysiological process of AD [31]. This study shows that in mice with AD induced by sommthelin-B knockout, vasoconstriction was reduced by about 30% [32,33,34]. Therefore, we hypothesized that in the process of vascular remodeling, on the one hand, the downregulation of MED12 reduced the total number of VSMC in the blood vessel walls, causing vessel walls to thin. On the other hand, a decrease in the proportion of synthetic VSMC reduced the synthesis and secretion of the extracellular matrix. Thus, the elasticity and toughness of the vascular wall were reduced, leading to the thinning of the arterial wall and participating in the process of vascular remodeling in diseases such as aortic dissection. 

Mediator Complex Subunit 12 (MED12) is a subunit of the Mediator Complex, which exerts its effects by influencing the transcriptional regulation process mediated by RNA polymerase II. Molecular genetic studies have demonstrated that MED12 gene mutations can lead to inherited diseases, with some patients presenting phenotypes such as vascular structural abnormalities (such as aneurysms [35]), while somatic MED12 mutations result in abnormal proliferative phenotypes, such as uterine leiomyoma [36] and fibroadenoma [37]. These results suggest that MED12 is an important gene involved in cell growth, proliferation, differentiation, and tissue and organ development. At present, there are many studies focusing on tumor diseases. Research on the involvement of MED12 in vascular diseases is still in the preliminary stage, and there are still gaps in the research. The basic pathological change of AD is the degeneration of middle aorta. Matrix metalloproteases (MMPs) are major enzymes that degrade extracellular matrix and basement membrane components of the aortic wall, so they play an important role in the development of AD. Previous studies have shown that the expression of MMP-9 and MMP-12 is increased in AD patients, and MMP-12 can degrade a broad spectrum of ECM components. Increased MMP-12 activity from inflammatory macrophages is also associated with abdominal aortic aneurysms, atherosclerosis, and emphysema [38]. In agreement with this, several studies reported an increased expression and activity of MMP-1, MMP-2, MMP-3, MMP-9, and MMP-12 in aortic aneurysm tissues [39]. In the skin of patients affected by severe chronic venous insufficiency (CVI), an elevated gene expression for MMP-1, MMP-2, MMP-9, and MMP-13 has been demonstrated. FXIII was able to block the urokinase-plasminogen activator (u-PA) plasmin pathway of MMP activation and thus improve the healing process [40]. Based on the above information, we found that MMP-9 is the shared genetic landscape. Interestingly, CDK8 stimulates expression of MMP-9 in human colon cancer cells via Wnt/β-catenin-driven transcription [41], while MED12 plays an important role in regulating and mediating the function of CDK8 kinase module, and the activation of CDK8 kinase also requires the participation of MED12 [42,43,44]. Our results show that MED12 is closely related to AD, and it is preliminarily clear that MED12 can affect the phenotype, proliferation, and aging process of vascular smooth muscle cells via the TGFβ signaling pathway. This study provides a new idea for exploring the mechanism of AD and a potential new target for the prevention and treatment of diseases related to vascular remodeling.

Current studies have shown that VSMC senescence is involved in the formation of arterial plaques and that VSMCs isolated from the lesion site show reduced proliferation and premature senescence in vitro. In vivo, the senescence VSMCs reduced the plaque’s self-repair ability and appeared vulnerable. Moreover, senescence VSMACs synthesized and secreted inflammatory cytokines (such as IL6) to promote plaque growth and overexpress chemokines (such as CCl2), adhesion molecules (such as ICAM-1), and immune receptors (such as TLR4) to form a pro-inflammatory environment that induces inflammatory cells into the plaque or promotes the proliferation, differentiation, and migration of surrounding cells [45]. The results of β-galactosidase staining show that inhibiting the expression of MED12 accelerates the aging of MOVAS, and the expression of P21 was significantly increased after the downregulation of MED12, suggesting that MED12 may promote the senescence of MOVAS by regulating the expression of p21. Recent studies reported that the accumulation of SM22α in VSMCs inhibits the degradation of TP53 and promotes the expression of P21, eventually causing cell senescence [46]. Therefore, the downregulation of MED12 may lead to the high expression of SM22α and participate in the senescence of VSMC.

In our study, the expression of MED12 in MOVAS decreased after incubation with TGFβ1, while the expression of TGFβR2 and the activity of the classical Smad signaling pathway were not significantly changed, but the activity of the ERK signaling pathway was significantly enhanced. This is different from the results reported by Huang et al., that MED12 inhibited the glycosylation of TGFβR2 and was unable to attach to the cell membrane. The downregulation of MED12 increased the amount of mature TGFβR2 on the cell membrane and enhanced the activity of the classical pathway involving downstream Smads and the nonclassical signaling pathway involving ERK protein [47]. It is suggested that MED12, as an inhibitor of the TGFβ1 signaling pathway in MOVAS, and the downregulation of MED12 enhanced the activity of the nonclassical TGFβ1 signaling pathway. TGFβ1, on the other hand, inhibited the phenotype transformation and proliferation of MOVAS. Sunil K et al. found that TGFβR2 protein expression was decreased and its downstream classical pathways and nonclassical pathways such as ERK were inhibited after knocking out MED12 in uterine leiomyoma cell lines [48], which was not completely consistent with the results of Huang et al. and our studies. Serial analysis of gene expression (SAGE) of MED12 was performed using the GeneCards database (http://www.genecards.org, accessed on 21 January 2020), and the results also showed that there were differences in the mRNA expression of MED12 in different tissues, suggesting that MED12 has different biological functions in different cells.

The involvement of MED12 in research on vascular remodeling-related diseases is still in the preliminary stage, and there are still gaps in the research work. First, although the results suggest that the MED12 protein mediates the TGFβ signaling pathway, its function in this pathway is still unclear, and the detailed molecular mechanism of TGFβ1 affecting MED12 must be further studied. Secondly, we only observed decreased proliferation, inhibited phenotypic conversion, and accelerated senescence of MOVAS after inhibition of MED12 at the cellular level. However, blood vessels are a complex organic whole composed of many types of cells, and there are complex interactions between all types of cells such that they are not independent of each other. Therefore, in vivo experiments should be conducted in subsequent studies to observe the effects of vascular remodeling by specific knockdown of MED12 in VSMC. In addition, MED12 will form the CDK8 kinase module with MED13, CYCC, and CDK8 and then combine with the intermediary complex to regulate gene transcription. Therefore, according to the current results, it is not clear whether MED12 needs to be integrated into the CDK8 kinase module when it plays a role in VSMC.

## 5. Conclusions

In this study, we observed decreased expression of MED12 in the aortae of AD patients and AD mice, which indicates that MED12 might participate in the pathological process of AD. Further experiments revealed that the downregulation of MED12 inhibited the proliferation of MOVAS and promoted cellular senescence through the TGFβ signaling pathway. Notably, the decreased MED12 promotes the contractile phenotype in MOVAS. This study provides a potential new target for the prevention and treatment of AD.

## Figures and Tables

**Table 1:** Primer sequences of target genes in RT-PCR.

Gene	Primer Sequences (5′-3′)
*Med12* (Mouse)	F: CCCGCCATGCCATCAAGAAGA R: CGTCGTCGCTTCTGCCCATCT
*Gapdh* (Mouse)	F: CATGGCCTTCCGTGTTCCTA R: CTGGTCCTCAGTGTAGCCCAA
*Actb* (Mouse)	F: ATCTGGCACCACACCTTC R: AGCCAGGTCCAGACGCA
*Mmp-2* (Mouse)	F: CCGCTGCGCTTTTCTCGAATC R: CCCAGGGTCCACAGCTCATCA
*Cdkn1a* (Mouse)	F: CGCTGGAGGGCAACTTCGTCTG R: GGGGAATCTTCAGGCCGCTCAG
*Pcna* (Mouse)	F: TCGAAGCACCAAATCAAGAGAAAGT R: ATTCACCCGACGGCATCTTTATTAC
*Tgfb1* (Mouse)	F: ACTGGAGTTGTACGGCAGTG R: GGGGCTGATCCCGTTGATTT
*Col4a1* (mouse)	F: AACAACGTCTGCAACTTCGC R: CTTCACAAACCGCACACCTG
*MED12* (Human)	F: GGCTGCCTGGCTCATTAAGAT R: GCCGGATTGCCACCTCTACAT
*GAPDH* (Human)	F: AGAAGGCTGGGGCTCATTTG R: AGGGGCCATCCACAGTCTTC
*ACTB* (Human)	F: CTGGGACGACATGGAGAAAA R: AAGGAAGGCTGGAAGAGTGC
*MMP-2* (Human)	F: GCTATGGACCTTGGGAGAA R: TGGAAGCGGAATGGAAAC

## Data Availability

No new data were created or analyzed in this study. Data sharing is not applicable to this article.
